# CD4^+^ and CD8^+^ TCRβ repertoires possess different potentials to generate extraordinarily high-avidity T cells

**DOI:** 10.1038/srep23821

**Published:** 2016-03-31

**Authors:** Munehide Nakatsugawa, Muhammed A. Rahman, Yuki Yamashita, Toshiki Ochi, Piotr Wnuk, Shinya Tanaka, Kenji Chamoto, Yuki Kagoya, Kayoko Saso, Tingxi Guo, Mark Anczurowski, Marcus O. Butler, Naoto Hirano

**Affiliations:** 1 Tumor Immunotherapy Program, Campbell Family Institute for Breast Cancer Research, Campbell Family Cancer Research Institute, Princess Margaret Cancer Centre, University Health Network, Toronto, ON M5G 2M9, Canada; 2 Department of Immunology, University of Toronto, Toronto, ON M5S 1A8, Canada; 3 Takara Bio, Inc., Kusatsu, Shiga 525-0058, Japan; 4 Department of Medicine, University of Toronto, Toronto, ON M5S 1A8, Canada

## Abstract

Recent high throughput sequencing analysis has revealed that the TCRβ repertoire is largely different between CD8^+^ and CD4^+^ T cells. Here, we show that the transduction of SIG35α, the public chain-centric HLA-A*02:01(A2)/MART1_27–35_ TCRα hemichain, conferred A2/MART1_27–35_ reactivity to a substantial subset of both CD8^+^ and CD4^+^ T cells regardless of their HLA–A2 positivity. T cells individually reconstituted with SIG35α and different A2/MART1_27–35_ TCRβ genes isolated from CD4^+^ or CD8^+^ T cells exhibited a wide range of avidity. Surprisingly, approximately half of the A2/MART1_27–35_ TCRs derived from CD4^+^ T cells, but none from CD8^+^ T cells, were stained by A2/MART1_27–35_ monomer and possessed broader cross-reactivity. Our results suggest that the differences in the primary structure of peripheral CD4^+^ and CD8^+^ TCRβ repertoire indeed result in the differences in their ability to form extraordinarily high avidity T cells which would otherwise have been deleted by central tolerance.

The αβ T-cell receptor (TCR), which is composed of a heterodimer of TCRα and β chains, recognizes antigenic peptides bound to major histocompatibility complex (MHC) class I or II molecules on the cell surface^1^. The TCRα and β chains possess three complementarity determining region (CDR) loops, which play an essential role in antigen recognition. The CDR1 and 2 loops are encoded within the germline Vα or β segment, and the hypervariable CDR3 region is determined by the junction of the spliced VJα and VDJβ gene segments involving random insertions and deletions of nucleotides^2^,^3^. As a consequence, the potential combinatorial diversity of the TCR repertoire exceeds 10^20^ 4. However, there are only 10^12^ T cells in the human body, and recent studies have estimated that there are <10^8^ different TCRs in the human naive T-cell repertoire^5^. The limited TCR repertoire must recognize many distinct peptide/MHC (pMHC) ligands to respond to a large array of foreign antigens expressed by any of a universe of pathogens and thus be cross-reactive^6^,^7^.

TCR signaling plays a central role in directing the developmental fate of thymocytes^8^. During thymocyte maturation, CD4 and CD8 coreceptor double-positive (DP) T cells mature and lead to coreceptor single-positive (SP) T cells in the thymus. DP thymocytes signaled by MHC class II-restricted TCRs differentiate into CD4^+^ SP T cells, whereas DP thymocytes signaled by MHC class I-restricted TCRs differentiate into CD8^+^ SP T cells. Typically, CD8^+^ and CD4^+^ T cells recognize peptides presented by MHC class I and class II molecules, respectively. However, numerous studies have reported that CD4^+^ T cells can recognize MHC class I-restricted antigens and CD8^+^ T cells recognize MHC class II-restricted antigens^9^,^10^,^11^,^12^,^13^,^14^. For example, TCR TRAV8/TRBV6 isolated from the alloreactive CD8^+^ T cell clone MBM15 recognizes both HLA–A2^+^ and HLA-DR1^+^ target cells^10^. TCR TRAV4/TRBV10-3 isolated from CD4^+^ tumor-infiltrating lymphocytes of a patient with metastatic malignant melanoma, TIL1383I, recognizes HLA–A2-restricted tyrosinase_368–376_ peptide in a CD8-independent manner^14^,^15^.

A chain-centric TCR hemichain can, on its own, determine MHC-restricted antigen specificity without requiring major contributions from the paired TCR counterchain^16^,^17^. We have recently reported that TCR chain centricity can be employed to produce a *de novo* antigen-specific T-cell repertoire, which can be used to isolate high-avidity antitumor T cells and their uniquely encoded TCRs^18^. Whereas a chain-centric TCR hemichain determines antigen specificity, the paired counterchain can regulate avidity over a broad range (>2 log orders) without compromising antigen specificity. We have also demonstrated that TCR chain centricity can be exploited to eliminate unwanted TCR cross-reactivity of antitumor TCRs^19^. TCR reactivity to target MHC/peptide complexes and cross-reactivity to unrelated MHC molecules are not inextricably linked and are separable at the TCR sequence level.

TCR sequences of a diverse T-cell repertoire can be analyzed directly by high-throughput sequencing^20^. Wang *et al.* reported that a substantial number of CDR3 sequences were overlapped between Th1, Th2, and Treg cells in CD4^+^ T cells, whereas a limited number of CDR3 sequences were found to be shared between CD8^+^ and CD4^+^ T cells in a healthy individual^21^. When the 100 most abundant CDR3β sequences were compared between CD8^+^ and CD4^+^ T cells, only 9 CDR3β clones were shared. Emerson *et al.* analyzed 13 million unique TCR sequences isolated from 42 adults, including healthy and multiple sclerosis patients, and identified sequence features in the CDR3β region of the TCR that distinguish CD4^+^ from CD8^+^ T cells^22^. Using high-throughput TCR sequence data, the authors estimated the CD4:CD8 ratio in unknown T cell samples from sequence data *in silico*. These results indicate that the peripheral CD8^+^ and CD4^+^ TCRβ repertoires have large differences in their primary structures. However, it is unknown how these differences in the TCRβ repertoire affect T cell avidity. Our aim here was to determine how the differences in the primary structure of peripheral CD8^+^ and CD4^+^ TCRβ repertoire influence the antigen-specificity and avidity of novel TCRs when paired with a fixed TCRα chain.

In the present study, we show that the differences in the primary structure of peripheral CD8^+^ and CD4^+^ TCRβ repertoire indeed result in differences in the ability to form extraordinarily high avidity T cells with broad cross-reactivity, which would otherwise have been deleted by central tolerance.

## Results

### When transduced with the chain-centric A2/MART1 TCRα hemichain SIG35α, both CD4^+^ and CD8^+^ T cells recognize A2/MART1 regardless of their A2 positivity

**Figure 1:**
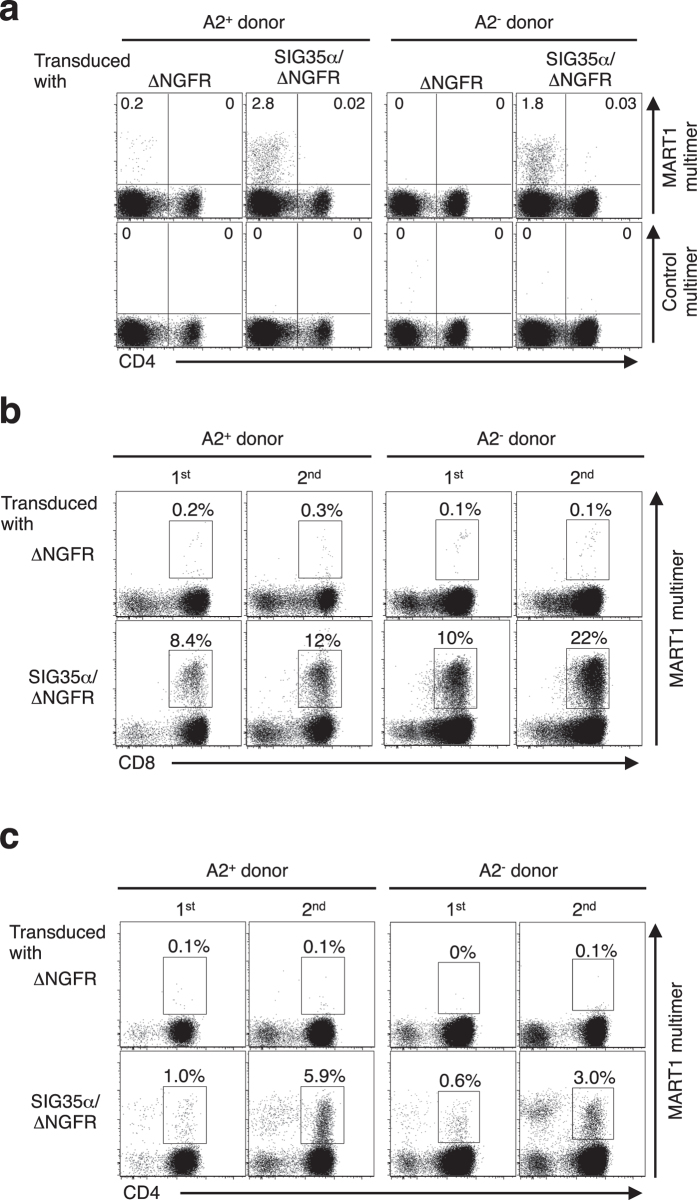
Both HLA–A2^+^ and A2^−^ peripheral CD4^+^ T cells as well as CD8^+^ T cells can recognize A2/MART1 when transduced with chain-centric SIG35α. (**a**) Peripheral T cells freshly isolated from HLA–A2^+^ and A2^−^ donors were retrovirally transduced with ΔNGFR alone or SIG35α/ΔNGFR and stained with A2/MART1 or control multimer in conjunction with anti-CD4 mAb and anti-NGFR mAb. The data shown are gated on ΔNGFR^+^ cells and representative of three independent experiments. (**b**,**c**) CD8^+^ T cells (**b**) or CD4^+^ T cells (**c)** transduced with ΔNGFR alone or SIG35α/ΔNGFR were purified using microbeads and stimulated with aAPC pulsed with A2/MART1 peptide once per week. Between stimulations, the T cells were supplemented with IL-2 (10 IU/ml) and IL-15 (10 ng/ml) every 3 days. The data from A2/MART1 multimer staining performed following the first and second stimulations are shown. The data shown are gated on ΔNGFR^+^ cells and representative of three independent experiments.

The chain-centric A2/MART1_27–35_ (hereafter A2/MART1) TCRα TRAV12-2/J35 hemichain, clone SIG35α, was isolated from multiple A2/MART1-specific CD8^+^ T-cell clones expressing different clonotypic TCRβ chains^16^,^17^. We investigated whether endogenous TCRβ chains expressed in CD4^+^ T cells recognize A2/MART1 when paired with SIG35α. Peripheral T cells from 2 donors, one HLA–A2^+^ and one A2^−^, were transduced with SIG35α alone and stained with A2/MART1 multimer (Fig. 1a). To distinguish A2/MART1 T cells derived from untransduced versus transduced T cells, the SIG35α gene was fused to the ΔNGFR gene by the F2A sequence. SIG35α^+^ A2/MART1 multimer^+^ CD4-negative T cells, i.e. CD8^+^ T cells, were detected as we reported previously^18^. However, A2/MART1 multimer^+^ CD4-positive T cells were not detected after transduction with SIG35α (Fig 1a).

Previously, we reported a series of human cell-based artificial antigen-presenting cells (aAPC), which can expand *in vitro* antigen-specific CD4^+^ and CD8^+^ T cells and polyclonal CD3^+^ T cells^23^,^24^,^25^,^26^,^27^. When exogenously pulsed with wild-type A2/MART1 peptide, aAPC stimulated SIG35α-transduced A2/MART1 CD8^+^ T cells from both A2^+^ and A2^−^ donors as reported elsewhere (Fig. 1b)^18^. Moreover, although the multimer positivity was substantially lower, the aAPC successfully expanded SIG35α^+^ A2/MART1 CD4^+^ T cells in a similar manner (Fig. 1c). These results suggest that a subset of endogenous TCRβ chains, not only in CD8^+^ T cells but also in CD4^+^ T cells, can recognize A2/MART1 when paired with SIG35α and that the frequency of such TCRβ chains seems lower in CD4^+^ T cells than in CD8^+^ T cells.

### SIG35α predominantly pairs with TRBV2, 5-1, and 27 TCRβ chains to recognize A2/MART1 in CD4^+^ T cells, and their CDR3β sequences are highly heterogeneous and unique

**Figure 2:**
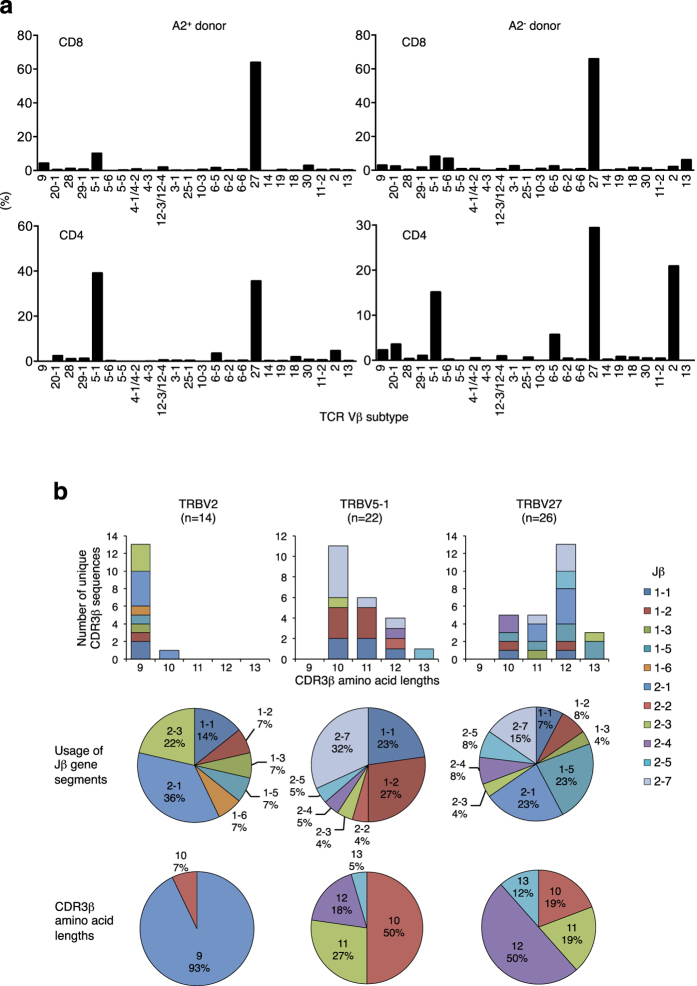
SIG35α predominantly pairs with TRBV2, 5–1, and 27 TCRβ chains with highly heterogeneous and unique CDR3β regions to recognize A2/MART1 in CD4^+^ T cells. (**a**) SIG35α/ΔNGFR-transduced peripheral CD8^+^ (top) and CD4^+^ (bottom) T cells from HLA–A2^+^ and A2^−^ donors were stimulated with aAPC pulsed with A2/MART1 peptide twice and stained with anti-NGFR mAb, A2/MART1 multimer, mAbs for TCR Vβ subtypes, and anti-CD8 or CD4 mAb. The percentage of SIG35α^+^ A2/MART1 multimer^+^ CD8^+^ (top) and CD4^+^ (bottom) T cells expressing each Vβ subtype is shown. The data shown are gated on ΔNGFR^+^ A2/MART1 multimer^+^ cells and representative of two independent experiments. The percentage of overall SIG35α-transduced T cells expressing each Vβ subfamily is shown in Supplementary Fig. 2. (**b**) SIG35α/ΔNGFR-transduced CD4^+^ T cells from the 2 donors were stimulated with aAPC pulsed with A2/MART1 peptide. ΔNGFR^+^ A2/MART1 multimer^+^ CD4^+^ T cells were collected by fluorescence-activated cell sorting (>99% purity), and their TRBV2, 5–1, and 27 CDR3β regions were amplified by PCR and sequenced after cloning. The number of unique CDR3β sequences (top), the relative usage of Jβ gene segments (middle), and the CDR3β amino acid lengths (bottom) are depicted separately for TRBV2 (left), TRBV5–1 (center) and TRBV27 (right).

As reported previously, SIG35α expressed in CD8^+^ T cells predominantly paired with TRBV27 TCRβ chains to recognize A2/MART1 in both A2^+^ and A2^−^ donors (Fig. 2a, top)^18^. Greater than 10^2^ clonotypic TRBV27 TCRβ genes were isolated from SIG35α^+^ A2/MART1 multimer^+^ CD8^+^ T cells and they were highly diverse in CDR3β sequences, Jβ usage, and amino acid lengths (Supplementary Fig. 1, Supplementary Table 1).

In contrast, SIG35α paired with TRBV2 and TRBV5-1 in addition to TRBV27 TCRβ chains to recognize A2/MART1 in CD4^+^ T cells in both A2^+^ and A2^−^ donors (Fig. 2a, bottom). Note that SIG35α was indeed transduced to CD4^+^ and CD8^+^ T cells expressing virtually all Vβ genes tested (Supplementary Fig. 2). To assess the CDR3β heterogeneity of the TRBV2, 5-1, and 27 genes paired with SIG35α for A2/MART1 reactivity, the TRBV2, 5-1, and 27 TCRβ genes were molecularly cloned from SIG35α^+^ CD4^+^ A2/MART1 multimer-positive cells from the two donors. We isolated a total of 14 TRBV2, 22 TRBV5-1 and 26 TRBV27 independent clonotypic TCRβ chains (Fig. 2b, Supplementary Table 2). Sequence analysis of the CDR3β region revealed the cloned TCRβ genes to be highly heterogeneous. All Jβ subfamilies except for Jβ1–4 and 2–6 were utilized. The length of CDR3β ranged from 9 to 13 amino acids. No clonotypic TCRβ gene was shared between the two donors (Supplementary Table 3). Additionally, there was no shared CDR3β sequence among the 174 TRBV27 clonotypes isolated from CD8^+^ T cells and 26 TRBV27 clonotypes isolated from CD4^+^ T cells (Supplementary Table 1, Supplementary Table 2). These results indicate that the CDR3β region sequences encoded by the *de novo*-generated CD4^+^ and CD8^+^ A2/MART1 T cells were both highly heterogeneous and unique.

### The avidity range of T cells individually reconstituted with the cloned TCRβ genes and SIG35α is broad

**Figure 3:**
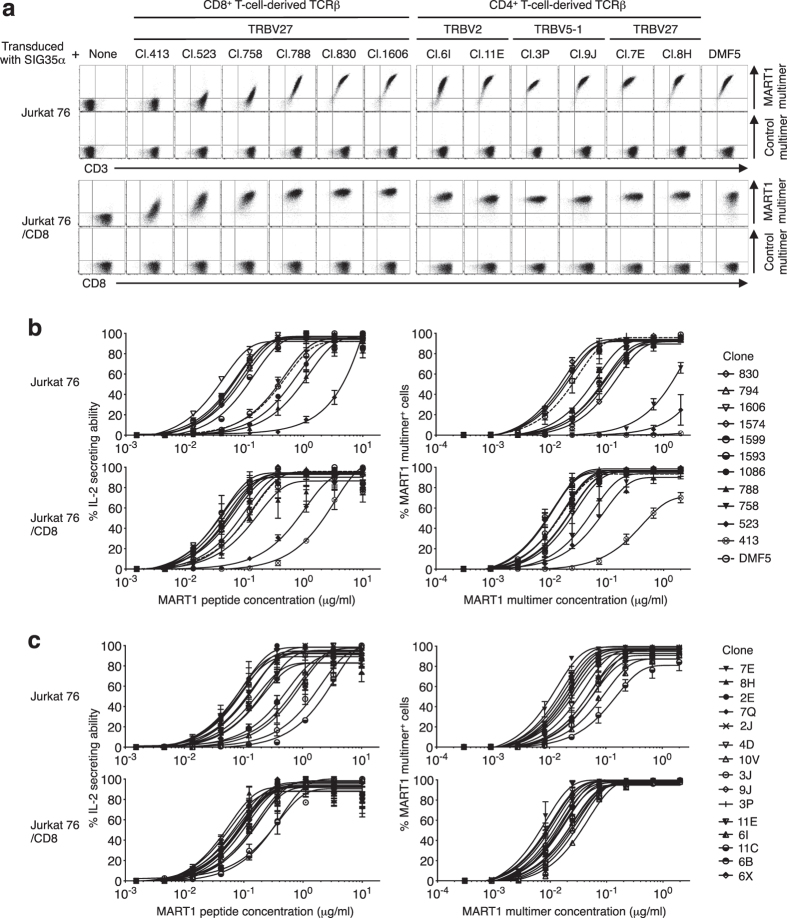
The avidity range of T cells individually reconstituted with the cloned TCRβ genes along with SIG35α is broad. Jurkat 76 cells, which lack the expression of CD8 and endogenous TCRs, were retrovirally transduced with CD8α and CD8β to produce Jurkat 76/CD8^18^. Jurkat 76 or Jurkat 76/CD8 cells were individually transduced with eleven clonotypic TRBV27 TCRβ chains cloned from SIG35α^+^ A2/MART1 CD8^+^ T cells or five clonotypic TCRβ chains each for TRBV2, TRBV5-1 and TRBV27 cloned from SIG35α^+^ A2/MART1 CD4^+^ T cells along with SIG35α or DMF5αβ chains. (**a**) All Jurkat 76 or Jurkat 76/CD8 transfectants were stained with 2 μg/ml A2/MART1 or control multimer along with anti-CD3 mAb or anti-CD8 mAb. Data for multimer staining of 6 representative CD8^+^ T-cell-derived TCRβ transfectants, 6 representative CD4^+^ T-cell-derived TCRβ transfectants, and DMF5 TCR transfectants in the presence or absence of CD8 coreceptor expression are shown. Data for multimer staining of the remaining transfectants are shown in Supplementary Fig. 3a,b. (**b**,**c**) Functional avidity of all CD8^+^ T-cell-derived TCRβ transfectants and the DMF5 TCR transfectant (**b**) and CD4^+^ T-cell-derived TCRβ transfectants (**c**) in the presence or absence of CD8 coreceptor expression are depicted as % IL-2 secreting ability as determined by IL-2 ELISPOT assay using T2 cells pulsed with graded concentrations of wild-type A2/MART1 peptide as stimulator cells (left). Structural avidity of the same transfectants is shown as multimer staining percentage determined by staining with graded concentrations of A2/MART1 multimer (right). All data shown are representative of three independent experiments.

To evaluate the avidity of SIG35α^+^ A2/MART1 T cells, we randomly selected five clonotypic TCRβ chains for each TRBV2, TRBV5-1 and TRBV27 cloned from SIG35α^+^ A2/MART1 CD4^+^ T cells and randomly selected eleven clonotypic TRBV27 TCRβ chains cloned from SIG35α^+^ A2/MART1 CD8^+^ T cells. These clonotypic TCRβ genes were individually reconstituted along with SIG35α on CD8^−^ TCR^−/−^ Jurkat 76 T cells in the presence or absence of CD8αβ (Fig. 3a, Supplementary Fig. 3a,b). All transfectants, including the one expressing A2/MART1 TCR, clone DMF5 (hereafter DMF5), demonstrated comparable surface CD3 expression, suggesting that the transduced TCR genes were expressed at equivalent levels. DMF5 is a naturally occurring A2/MART1 TCR with affinity among the highest that has been used in TCR gene transfer clinical trials^28^. Two of the eleven CD8^+^ T-cell-derived TCRβ transfectants, the ones expressing cl.413 and 523, were not stained by A2/MART1 multimer in the absence of CD8 coreceptor expression, suggesting low structural avidity (Fig. 3a). The remaining 9 transfectants were stained by A2/MART1 multimer even in the absence of CD8, suggesting high structural avidity. All CD8^+^ T-cell-derived TCRβ transfectants were stained by A2/MART1 multimer in the presence of CD8 coreceptor expression. In contrast, all 15 CD4^+^ T-cell-derived TCRβ transfectants were stained by A2/MART1 multimer even in the absence of CD8 coreceptor expression, indicating high structural avidity.

We then comprehensively compared the structural and functional avidity of all Jurkat 76 transfectants individually expressing A2/MART1 TCRβ chains derived from CD4^+^ and CD8^+^ T cells along with SIG35α (Fig. 3b,c). Structural avidity, expressed as the EC_50_ in μg/ml, was defined as the concentration of A2/MART1 multimer required to achieve 50% of the maximal staining intensity. Similarly, functional avidity, shown as the EC_50_ in μg/ml, was determined by the concentration of A2/MART1 peptide required to achieve 50% of the maximal response in cytokine secretion analysis. Data for the structural and functional avidity of all transfectants in the absence or presence of CD8 are summarized in Table 1. These transfectants demonstrated a wide range of both structural and functional avidity, which was augmented by the CD8 coexpression. Structural avidity measured using a multimeric complex as well as functional avidity were comparable between transfectants expressing A2/MART1 TCRβ derived from CD4^+^ and CD8^+^ T cells (Supplementary Fig. 3c).

### When paired with SIG35α, A2/MART1 TCRβ genes isolated from CD4^+^ T cells demonstrate broader cross-reactivity compared with those from CD8^+^ T cells

**Figure 4:**
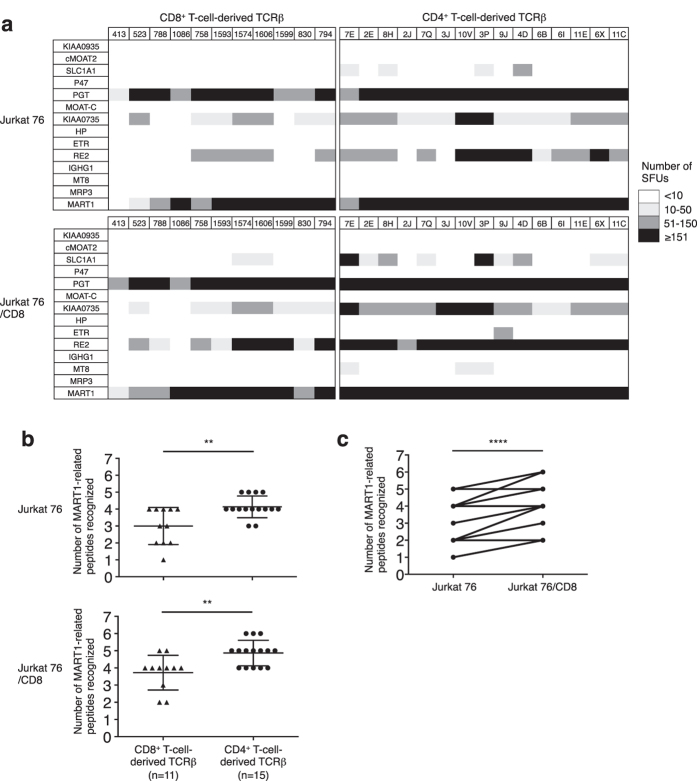
When paired with SIG35α, A2/MART1 TCRβ genes isolated from CD4^+^ T cells possess broader cross-reactivity compared with those from CD8^+^ T cells. (**a**) Peptide recognition was assessed using T2 cells loaded with 10 μg/ml of MART1-related peptides as stimulator cells in IL-2 ELISPOT assays. Thirteen MART1-related peptides are shown in Supplementary Table 4^29^. Fifty-thousand Jurkat 76 or Jurkat 76/CD8 cells expressing one of 11 clonotypic CD8^+^ T cell-derived TCRβ chains or 15 clonotypic CD4^+^ T-cell-derived TCRβ chains along with SIG35α were used as responder cells. The results are presented as grayscale plots. The vertical axis indicates MART1-related peptides including wild-type MART1 peptide, and the horizontal axis indicates the reconstituted TCRβ chains. SFUs, spot-forming units. (**b**) The number of MART1-related peptides recognized was compared in Jurkat 76 (top) and Jurkat 76/CD8 (bottom) transfectants expressing CD8^+^ or CD4^+^ T-cell-derived TCRβ chains. The data represent the means ± SD in each group. **p < 0.01, two-tailed Welch’s t test. (**c**) The number of MART1-related peptides recognized was compared in all Jurkat 76 transfectants with or without CD8 coexpression. The data represent the means ± SD in each group. ****p < 0.0001, two-tailed paired t test. All data shown are representative of two independent experiments.

Cross-reactivity, commonly observed in TCR, is considered to be a general propensity of TCRs^6^,^7^. We therefore evaluated the cross-reactivity of reconstituted A2/MART1 TCRs to MART1-related peptides selected by positional scanning synthetic peptide library screening (Supplementary Table 4)^29^. Jurkat 76 or Jurkat 76/CD8 A2/MART1 TCR transfectants recognized one to six MART1-related peptides, including wild-type A2/MART1 peptide, in the context of HLA–A2 (Fig. 4a). Interestingly, the 15 transfectants expressing CD4^+^ T-cell-derived TCRβ genes recognized significantly more MART1-related peptides compared with the 11 transfectants expressing TCRβ chains isolated from CD8^+^ T cells regardless of CD8 coexpression (Fig. 4b). We also investigated whether CD8 coexpression enhances the observed TCR cross-reactivity^30^. As shown in Fig. 4c, the number of MART1-related peptides recognized by all Jurkat 76 TCR transfectants was significantly higher in the presence of CD8 coexpression than in its absence. These results show that CD4^+^ T-cell-derived TCRβ transfectants possess broader cross-reactivity compared with CD8 TCRβ transfectants.

### Extremely high-avidity T cells expressing CD4^+^ T cell-derived TCRβ chains, which can be stained by an A2/MART1 monomer complex, possess broader cross-reactivity

**Figure 5:**
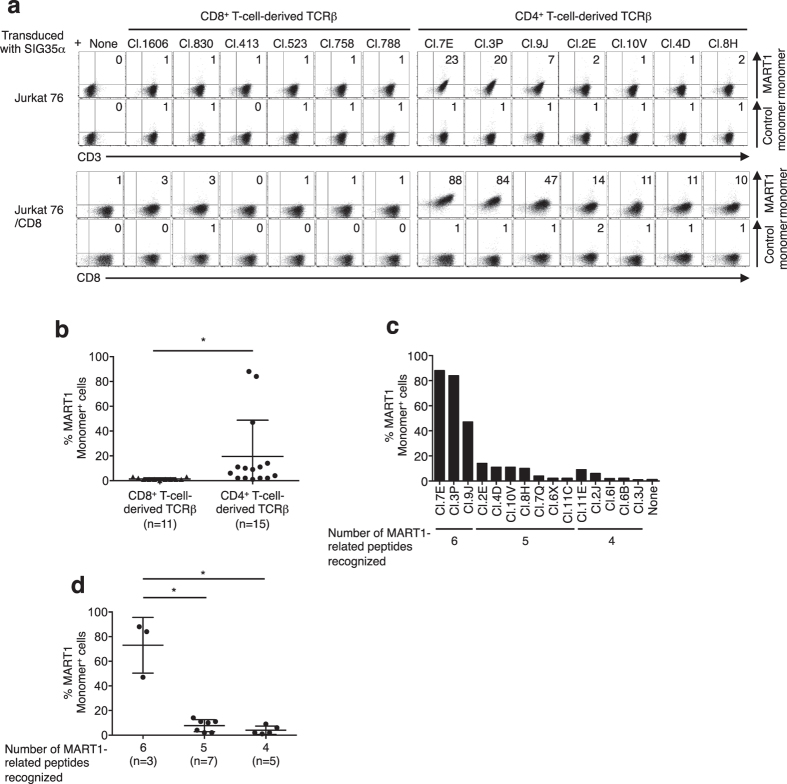
Extremely high-avidity T cells expressing CD4^+^ T cell-derived TCRβ chains, which can be stained by an A2/MART1 monomer complex, possess broader cross-reactivity. (**a**) Jurkat 76 and Jurkat 76/CD8 transfectants were stained with 50 μg/ml non-multimerized A2/MART1 or control monomer along with anti-CD3 mAb or anti-CD8 mAb. Data for monomer staining of 6 representative CD8^+^ T-cell-derived TCRβ transfectants and 7 representative CD4^+^ T-cell-derived TCRβ transfectants are shown. Data for monomer staining of the remaining transfectants are shown in Supplementary Fig. 4a,b. (**b**) A2/MART1 monomer positivity was compared in Jurkat 76/CD8 transfectants expressing CD8^+^ or CD4^+^ T-cell-derived TCRβ chains. The data represent the means ± SD in each group. *p < 0.05, two-tailed Welch’s t test. (**c**) A2/MART1 monomer positivity in Jurkat 76/CD8 transfectants expressing CD4^+^ T-cell-derived TCRβ chains is shown according to the number of MART1-related peptides recognized. (**d**) A2/MART1 monomer positivity was compared in Jurkat 76/CD8 transfectants expressing CD4^+^ T-cell-derived TCRβ chains that recognized the indicated number of MART1-related peptides. The data represent the means ± SD in each group. *p < 0.05, two-tailed Welch’s t test. The data shown are representative of three independent experiments.

Because of the low-affinity interaction between TCRs and peptide/MHC (pMHC) complexes, pMHC monomers cannot stain T cells in general. Therefore, various multimeric pMHC forms such as tetramers have been produced and utilized to stain cognate T cells^31^,^32^,^33^. It has been suggested that the measurement of structural avidity using pMHC multimers underestimates the difference in the structural avidity of T cells because of the avidity ‘bonus’ effect caused by the multivalency of pMHC multimers^31^,^34^. We investigated whether the reconstituted A2/MART1 T cells were sufficiently avid to be stained by a non-multimerized A2/MART1 monomer complex (Fig. 5a, Supplementary Fig. 4a,b). None of the 11 CD8^+^ T-cell-derived TCRβ transfectants was stained by A2/MART1 monomer. Surprisingly, approximately half (7 out of 15) of clonotypic TCRβ transfectants, which were isolated from SIG35α^+^ A2/MART1 CD4^+^ T cells, were clearly stained by A2/MART1 monomer in the presence of CD8 coreceptor. The 2 transfectants, clones 7E and 3P, were substantially stained even in the absence of CD8, suggesting that they possess extremely high avidity. The monomer positivity of CD4^+^ T-cell-derived TCRβ transfectants was significantly higher compared with that of CD8^+^ T-cell-derived TCRβ transfectants (Fig. 5b). These results indicate that T cells reconstituted with naturally occurring TCRβ chains endogenously expressed in CD4^+^ T cells can be sufficiently avid to be positive for A2/MART1 monomer when paired with SIG35α.

We then evaluated the correlation between the cross-reactivity and monomer positivity of the A2/MART1 TCR transfectants. Interestingly, the three transfectants, clones 7E, 3P, and 9J, that recognized 6 MART1-related peptides showed significantly higher monomer positivity compared with the remaining 12 clones that cross-reacted with 4 or 5 A2/MART1-related peptides (Fig. 5c,d). These results suggest that highly avid A2/MART1 T cells, which express TCRβ genes derived from CD4^+^ T cells and can be stained by an A2/MART1 monomer complex, possess broader cross-reactivity.

### Highly avid CD8^+^ T cells lose target cell specificity

**Figure 6:**
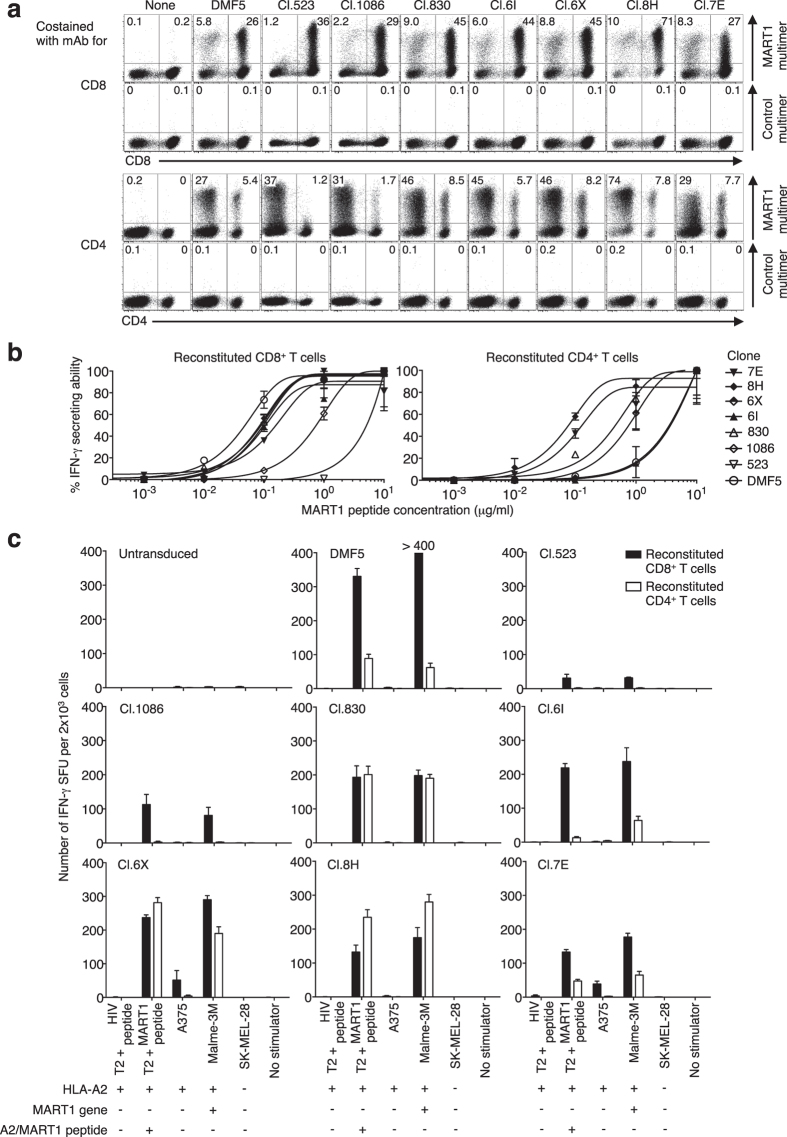
Highly avid CD8^+^ T cells lose target cell specificity. (**a**) Peripheral T cells were reconstituted with TCRβ (cl. 523, 1086, 830, 6I, 6X, 8H, or 7E) genes along with SIG35α, or with DMF5 TCR, and stained with A2/MART1 or control multimer in conjunction with anti-CD8 (top) or anti-CD4 mAb (bottom). (**b**) Reconstituted CD8^+^ or CD4^+^ T cells were purified using microbeads and used as responder cells in IFN-γ ELISPOT assays. Functional avidity values of reconstituted CD8^+^ (left) and CD4^+^ (right) T cells are depicted as % IFN-γ secreting ability as determined by IFN-γ ELISPOT assay using T2 cells pulsed with graded concentrations of A2/MART1 peptide as stimulator cells. Note that functional avidity for CD4^+^ T cell-expressing cl. 523 and 1086 with low reactivity could not be analyzed. (**c**) IFN-γ ELISPOT assays were performed using reconstituted CD8^+^ or CD4^+^ T cells as responder cells. T2 cells pulsed with 10 μg/ml A2/HIV control peptide or A2/MART1 peptide, and melanoma cells that did or did not express HLA–A2 and/or MART1, were used as stimulator cells. The data represent the means ± SD in each group and are representative of two independent experiments.

Based on the results above, we selected 3 clonotypic CD8^+^ T-cell-derived TCRβ (cl. 523, 1086 and 830) and 4 clonotypic CD4^+^ T-cell-derived TCRβ (cl. 6I, 6X, 8H and 7E) varying in their A2/MART1 reactivity. These TCRβ chains showed low to high avidity, and cross-reactivity when paired with SIG35α, and were individually reconstituted along with SIG35α on peripheral T cells. DMF5 was also reconstituted for comparison. Note that except for DMF5, the TCRα chain SIG35α was shared in all these T cells. Except for T cells expressing cl. 523, A2/MART1 multimer stained the transduced CD4^+^ T cells as well as CD8^+^ T cells (Fig. 6a). A2/MART1 multimer stained cl. 523-transduced CD8^+^ T cells, but did not stain the transduced CD4^+^ T cells, suggesting low structural avidity. CD8^+^ and CD4^+^ T cells were purified (97% or better purity) to analyze their function separately (Supplementary Fig. 5). We first evaluated the functional avidity of the transduced CD8^+^ and CD4^+^ T cells using IFN-γ ELISPOT analysis (Fig. 6b). Note that the analysis could not be performed for CD4^+^ T cell-expressing cl. 523 and 1086 with low reactivity. CD4^+^ T cells expressing cl. 7E, 8H, 6X or 830 possessed higher functional avidity compared with the DMF5 transfectant. Paradoxically, CD8^+^ T cells expressing cl. 7E, 8H, 6X or 830 possessed lower functional avidity than DMF5-transduced CD8^+^ T cells. CD8^+^ T cells expressing DMF5 possessed the highest functional avidity in CD8^+^ T cells. These results are in accordance with previous findings that high-affinity class I-restricted TCRs do not always confer high functional avidity to CD8^+^ T cells^35^,^36^,^37^. We next compared the A2/MART1 specificity of peripheral CD4^+^ and CD8^+^ T cells individually expressing the 8 different cognate TCRs. Except for those expressing cl. 523 and 1086, all CD4^+^ T cells recognized A2^+^MART1^+^ cells but not A2^+^MART1^−^ or A2^−^MART1^−^ cells. The CD4^+^ T cells expressing cl. 523 and 1086 recognized neither T2 cells loaded with MART1 peptide nor A2^+^ MART1^+^ cells, likely due to their low functional avidity. All CD8^+^ T cells, except for those expressing cl. 7E and 6X, recognized A2^+^ MART1^+^ target cells specifically. However, CD8^+^ T cells expressing cl. 7E and 6X, which were isolated from CD4^+^ T cells, recognized A2^+^ MART1-negative A375 cells, indicating that the CD8^+^ T cells expressing cl. 7E and 6X recognized naturally processed and presented peptide(s) other than the A2/MART1 peptide (Fig. 6c). The cross-reactivity of CD8^+^ T cells expressing cl. 7E and 6X to A2^+^ MART1^−^ cells was largely diminished when CD8 coengagement was blocked by anti-CD8 mAb (Supplementary Fig. 6). These results suggest that CD8^+^, but not CD4^+^ T cells transduced with CD4 TCRβ chains can have cross-reactivity to endogenously processed and presented peptide(s) distinct from cognate peptide possibly due to ‘excessive’ CD8 coreceptor function.

## Discussion

It has been shown that class I-restricted antigen-specific CD4^+^ T cells as well as CD8^+^ T cells are detected by peptide/HLA multimers in peripheral T cells^12^. Interestingly, A2/MART1 multimer^+^ CD4^+^ T cells isolated from patient samples expressed TRAV12-2, which is also predominantly expressed by A2/MART1 CD8^+^ T cells, suggesting that the TRAV12-2 CDR1/2 region plays a dominant role in A2/MART1 recognition^16^,^17^. We generated highly polyclonal A2/MART1-specific CD8^+^ and CD4^+^ T cells by transducing CD8^+^ and CD4^+^ T cells with a chain-centric TCRα hemichain, clone SIG35α, and analyzed their TCRβ sequences. There was no CDR3β clonotype shared between TCRβ chains isolated from SIG35α^+^ A2/MART1 CD8^+^ and CD4^+^ T cells (Supplementary Table 1, Supplementary Table 2). SIG35α^+^ A2/MART1 CD4^+^ T cells expressed TRBV2 and TRBV5-1 in addition to TRBV27 TCRβ chains (Fig. 2a). TRBV2 and TRBV5-1 TCRβ chains have not been frequently observed in A2/MART1-specific CD8^+^ T cells isolated from the periphery or tumor sites in contrast to TRBV27^16^,^17^,^24^,^38^. In addition, the Jβ1–3 gene segment, which is encoded by A2/MART1 monomer-positive cl. 7E, was isolated only from SIG35α^+^ A2/MART1 CD4^+^ T cells but not from the SIG35α^+^ A2/MART1 CD8^+^ T cells. These results suggest that TCRs expressed by peripheral CD4^+^ and CD8^+^ T cells differ not only in their primary structure but also in their potential to generate extremely high-avidity T cells.

The vast majority of TCRs have low affinity for pMHC^2^. Although a single pMHC complex can induce a detectable calcium signal via TCRs, a monomeric pMHC complex is generally unsuitable for staining T cells for flow cytometry analysis^39^. Therefore, multimeric forms such as dimers, tetramers, pentamers, and dextramers have been developed to stain antigen-specific T cells^31^,^32^,^33^.

We showed that a non-multimerized A2/MART1 monomer complex was able to stain approximately half (7/15) of the clonotypic TCRβ chains isolated from SIG35α^+^ A2/MART1 CD4^+^ T cells and paired with SIG35α (Fig. 5a). Surprisingly, at least two of the seven TCRβ chains were clearly positive (>10%) for monomer staining even in the absence of CD8 coreceptor. In contrast, none of the eleven clonotypic TCRs isolated from SIG35α^+^ A2/MART1 CD8^+^ T cells was stained by A2/MART1 monomer (Fig. 5a, Supplementary Fig. 4a). These results suggest that there exists a subset of TCRβ chains that are not shared by CD8^+^ and CD4^+^ T cells. SIG35α is a naturally occurring public A2/MART1 TCRα chain that has frequently been isolated from multiple individuals as reported previously^16^,^17^,^40^. Because all TCRβ genes reported in this study are naturally occurring and expressed in peripheral T cells, heterodimers consisting of SIG35α and isolated TCRβ chains can be expressed in thymocytes in theory. To the best of our knowledge, there has been no report of naturally occurring T cells that can be stained with a monomeric pMHC complex using a standard staining method. It has been reported that photocrosslinking technology allows for the successful staining of cognate T cells with monomeric pMHC^41^. Our results indicate that thymus-derived T cells can possess sufficiently high avidity to be stained by a pMHC monomer complex. Such extremely highly avid T cells are most likely deleted through negative selection in the thymus.

TCR cross-reactivity is an intrinsic characteristic of antigen-specific T cells^6^,^42^; it is a critical attribute of T cells that protects the host from any virus with mutations using a limited TCR repertoire. In fact, the degree of TCR cross-reactivity can affect disease control during infection with viruses such as HIV that have a high mutation rate^43^. In contrast, when TCRs cross-react with self-antigens, the cross-reactivity can lead to harmful autoimmune responses. In recent TCR gene therapy clinical trials, A2/MAGE-A3_112–120_ TCR- and A1/MAGE-A3_168–176_ TCR-transduced T cells recognized non-target self-antigens and caused off-target/off-tumor lethal toxicity^44^,^45^,^46^.

The use of a panel of high-affinity A2/NY-ESO-1_157–165_ TCRs, revealed that increased affinity is associated with a loss of target cell specificity of TCR gene-modified CD8^+^ T cells^47^. Similarly, a CD8^+^ hybridoma expressing a high-affinity TCR, clone m33α, which is a derivative of K^b^/SIY TCR clone 2C, demonstrated cross-reactivity to self-pMHC complexes^48^. We demonstrated that CD8^+^ T cells expressing cl. 7E and 6X, which possess broad cross-reactivity to predicted analogous peptides, indeed recognized naturally processed and presented self-peptides other than the target A2/MART1 peptide, whereas CD4^+^ T cells did not (Fig. 6c). As shown in Fig. 4c, CD8 coengagement enhances cross-reactivity^30^. These findings suggest that CD8 coengagement is unbeneficial or even detrimental for highly avid CD8^+^ T cells because it may make their undetectable intrinsic cross-reactivity overt, rendering them harmfully autoreactive (Supplementary Fig. 6).

In generating a T-cell graft for TCR gene therapy clinical trials, both CD8^+^ and CD4^+^ T cells are generally transduced with exogenous TCR genes. A recent study reported that CD8^+^ and CD4^+^ T cells expressing high-affinity anti-tyrosinase TCR, which is a CD8 coreceptor-independent TCR, were equally effective at mediating tumor regression *in vivo*^15^. Kranz and colleagues reported that the adoptive transfer of CD4^+^ T cells expressing affinity-enhanced class I-restricted TCRs inhibited tumor growth^49^. These studies indicate that CD4^+^ T cells expressing a high-affinity or coreceptor-independent TCR against a class I-restricted tumor antigen can recognize tumor cells and mediate tumor regression directly or indirectly.

We isolated at least 5 high-affinity A2/MART1 TCR clonotypes that recognized A2^+^ MART1^+^ tumor cells in a CD8-independent manner when expressed in CD4^+^ T cells (Fig. 6c). Interestingly, peripheral CD4^+^ T cells expressing any of the 4 TCRs possessed functional avidity higher than or similar to those expressing DMF5, whereas CD8^+^ T cells expressing DMF5 possessed the highest functional avidity (Fig. 6b). These results suggest that there is a difference in the optimal range of TCR affinity required for optimal T-cell function between TCR-transduced CD8^+^ and CD4^+^ T cells, which is significantly affected by CD8 coreceptor coengagement^50^. In addition, T cells expressing cl. 7E with the highest structural avidity did not possess the highest functional avidity (Fig. 6b, Table 1). These findings are consistent with previous studies demonstrating the existence of an affinity threshold, above which CD8^+^ T-cell function is not improved or reduced^35^,^36^,^37^. Stable high-affinity interactions between TCR:pMHC with long half-life inhibit one pMHC complex from serial TCR triggering, i.e., sequential binding and stimulation of multiple TCR molecules^51^. Paradoxically, as a result of improved serial TCR triggering, low-affinity interactions may stimulate T-cell activation more efficiently than stable long-lived interactions between high affinity TCRs and their cognate HLA/peptide complex^52^. Similar findings have also been observed in chimeric antigen receptor studies^53^.

All the TCR hemichain genes studied herein were isolated from peripheral T cells and are therefore naturally occurring. We observed that endogenous TCRβ chains from CD4^+^ T cells formed TCRs with extremely high affinity for A2/MART1 when paired with SIG35α TCRα, with higher likelihood than TCRβ from CD8^+^ T cells. This may be a consequence of different outcome of thymic selection. Since the avidity and likely cross-reactivity of A2/MART1-reactive TCRs toward other self A2/peptide complexes increases when co-expressed with CD8 (see Figs 3a,5a and 6c), CD8^+^ thymocytes that express these high affinity TCRs are more likely to be removed from the repertoire than CD4^+^ thymocytes expressing the same TCRs. Meanwhile, CD4^+^ thymocytes expressing such cross-reactive TCRs can be positively selected independently of A2, due to their appropriate affinity for MHC class II/peptide complexes. Our results suggest that the post-thymic CD4^+^ T-cell repertoire can serve as a source for the isolation of class I-restricted T cells with extremely high avidity, albeit with broad cross-reactivity. Peripheral CD4^+^ and CD8^+^ TCRβ repertoires are distinct not only in their primary sequence, as has been shown, but also in their capacity to generate thymically unselected T cells with extraordinarily high avidity.

## Methods

### Cells

Peripheral blood mononuclear cells (PBMCs) were obtained from healthy donors. University Health Network Research Ethics Board approval and appropriate informed consent were obtained. All experiments were carried out in accordance with guidelines approved by the University Health Network Biosafety Committee. Mononuclear cells were obtained by density gradient centrifugation (Ficoll-Paque PLUS; GE Healthcare). All donors were identified as positive or negative for HLA-A*02:01 (A2) by high-resolution HLA DNA typing (American Red Cross). K562 is an erythroleukemic cell line defective for HLA expression. T2 is an HLA–A2 positive T cell leukemia/B-LCL hybrid cell line. A375 (A2^+^, MART1^−^), Malme-3M (A2^+^, MART1^+^), and SK-MEL-28 (A2^−^, MART1^+^) are melanoma cell lines. PG13 is a gibbon ape leukemia virus-packaging cell line and Phoenix Eco is an ecotropic packaging cell line. Jurkat 76 is a T cell leukemic cell line lacking TCR and CD8 expression (a gift from Dr. M. Heemskerk)^54^. Jurkat 76 was cultured in RPMI 1640 supplemented with 10% FCS and gentamicin (Life Technologies, Carlsbad, CA). All cell lines except for Jurkat 76 were obtained from American Type Culture Collection (ATCC, Manassas, VA) and cultured according to the provided instructions. All cells were routinely checked for the presence of mycoplasma contamination using the polymerase chain reaction-based Mycoplasma Detection Kit from ATCC.

### cDNAs

The codon-optimized A2/MART1 TCR gene (clone SIG35α) was produced by Life Technologies (Carlsbad, CA) according to the published sequence^16^,^17^. SIG35α was fused with a truncated version of human nerve growth factor receptor (ΔNGFR) via an optimized intervening sequence consisting of a furin cleavage site, an SGSG spacer sequence, and an F2A sequence^55^. The A2/MART1_27–35_ TCR (clone DMF5) gene was kindly provided by Dr. S. Rosenberg (NIH/NCI, Bethesda, MD). Full-length cDNAs encoding TRBV2, 5-1 and 27 TCRβ genes were molecularly cloned via RT-PCR using the TCRβ gene-specific forward primers, and reverse primers for Cβ1 and Cβ2 constant regions. Forward primers were TRBV2 5′-ATCCCAGTGTGGTGGTAGGGAATTCGCCACCATGGATACCTGGCTCGTATGC-3′, TRBV5-1 5′-ATCCCAGTGTGGTGGTACGGGAATTCGCCACCATGGGCTCCAGGCTGCTCTGTTGG-3′ and TRBV27 5′-ATCCCAGTGTGGTGGTACGGGAATTCTGCCATGGGCCCCCAGCTCCTTGGC-3′. Reverse primers were Cβ1 5′-ATCGTCGACCACTGTGCTGGCGGCCGCTCGAGTTCCAGGGCTGCCTTCAGAAATCC-3′) and Cβ2 5′-GACCACTGTGCTGGCGGCCGCTCGAGCTAGCCTCTGGAATCCTTTCTCTTGACCATTGC-3′). The PCR products were generated using 1 U/50 μl of Phusion high-fidelity DNA polymerase (New England Biolabs, Ipswich, MA), 0.5 μM primers, and 200 μM dNTPs by incubation at 98 °C for 30 seconds, followed by 35 amplification cycles of 98 °C for 10 seconds, 60 °C for 30 seconds, and 72 °C for 15 seconds. The cDNAs were cloned into the pMX vector to transduce all cell lines and primary human T cells as reported previously^18^.

### Analysis of TCRβ sequences

Nucleotide sequencing was performed using a pMX vector-specific forward primer, 5′-TGGATACACGCCGCCCA-3′ and reverse primer, 5′-CCCTTTTTCTGGAGACTAAAT-3′ at the Centre for Applied Genomics. The Hospital for Sick Children (Toronto, Canada). TCRα and β gene allele names are in accordance with IMGT unique gene nomenclature (http://www.imgt.org/). Jβ gene usage and CDR3 regions were determined using the IMGT/V-QUEST tool^56^.

### Peptides

The peptides used were A2-restricted wild-type MART1_27–35_ (_27_AAGIGILTV_35_) and HIV pol_476–484_ (A2/HIV) (_476_ILKEPVHGV_484_) peptides. A2/HIV pol_476–484_ peptide was always used as a control peptide. The thirteen MART1-related peptides used were KIAA0935_450–459_ (_450_RVTDEAGHPV_459_), cMOAT2_1353–1362_ (_1353_NVADIGLHDV_1362_), SLC1A1_289–298_ (_289_VLTGLAIHSI_298_), P47_317–326_ (_317_RISDIRLFIV_326_), prostaglandin transporter_178–187_ (_178_LLAGIGTVPI_187_), MOAT-C_1257–1266_ (_1257_RISDIGLADL_1266_), KIAA0735_206–215_ (_206_LISGIGIGGA_215_), hypothetical 20 kD protein_3–12_ (_3_RISAIILHPN_12_), endothelin-1 receptor_192–201_ (_192_RVQGIGIPLV_201_), G-protein coupled receptor RE2_364–373_ (_364_RITDLGLSPH_373_), IGHG1_102–111_ (_102_RLSELAIFGV_111_), monocarboxylate transporter 8_179–188_ (_179_AVAFIGLHTS_188_), and MRP3_1353–1362_ (_1353_NVADIGFHDV_1362_)^29^. Synthetic peptides were obtained from Genscript (Piscataway, NJ). Throughout the study, wild-type but not heteroclitic A2/MART1 peptide was utilized for the expansion and functional analysis of T cells.

### Transfectants

Jurkat 76 was transduced with CD8α and CD8β cDNAs to generate Jurkat 76/CD8 as reported previously^18^. Jurkat 76 or Jurkat 76/CD8 transfectants were further transduced with individual TCRβ genes along with SIG35α, and the transfectants were purified using anti-CD3 Microbeads (Miltenyi Biotec). K562-based artificial antigen-presenting cells (aAPC) stably express mutated HLA–A2 in conjunction with CD80 and CD83^23^,^26^. The mutated HLA–A2 molecules bear two amino acid substitutions at positions 227 and 228 that abrogate the interaction with CD8^57^. aAPC was engineered to constitutively secrete IL-21 to enable T cell expansion^26^,^58^. PG13-based packaging cells were generated by first transfecting Phoenix Eco cells with 20 μg of DNA for each construct using the TransIT-293 reagent (Mirus Bio, Madison, WI). PG13 cells were then transduced with supernatant from Phoenix Eco cells. PG13-derived retrovirus supernatant was utilized to transduce TCR genes into Jurkat 76, Jurkat 76/CD8, and human primary T cells as reported previously^18^. A retroviral vector encoding ΔNGFR alone was employed as a control vector.

### Expansion of TCR gene-modified T cells in an HLA–A2-restricted peptide-specific manner

Peptide-specific T cells were expanded using an aAPC as described previously^23^,^24^. PBMCs were isolated from healthy volunteers and stimulated with 50 ng/ml anti-CD3 mAb (clone OKT3) in the presence of 100 IU/ml human IL-2 (Novartis) 3 days before transduction. Activated T cells were retrovirally transduced with TCR genes by centrifuging 1 hour at 1,000 g at 32 °C. Following transduction, CD4^+^ or CD8^+^ T cells were purified using anti-CD4 or anti-CD8 Microbeads (Miltenyi Biotec) and plated at 2 × 10^6^ cells/well in RPMI 1640 supplemented with 10% human AB serum. The stimulator aAPC was pulsed with 10 μg/ml A2-restricted wild-type MART1_27–35_ for 6 hours at room temperature. The aAPC was then irradiated at 200 Gy, washed, and added to the responder T cells at a responder to stimulator ratio of 20:1. Starting the next day, 10 IU/ml IL-2 (Novartis) and 10 ng/ml IL-15 (Peprotech) were added to the cultures every 3 days. T cells were harvested, counted, and restimulated every week. T cell analysis was performed one day prior to or on the day of restimulation.

### Flow cytometry analysis

The following mAbs recognizing the indicated antigens were used: CD4 (IM2636U, 1:100, Beckman Coulter), CD8 (IM2638U, 1:100, Beckman Coulter); NGFR (345104, 1:40, BioLegend); CD3 (300440, 1:100, BioLegend) and Biotin (409004, 1:100, BioLegend). Assessment of TCR Vβ subfamily usage was performed using a Beta Mark TCR Vβ Repertoire Kit (IM3497, 1:4, Beckman Coulter) as reported previously^24^. The surface molecule staining and subsequent flow cytometry analysis were performed as described elsewhere^59^.

### HLA/peptide multimer staining

Biotinylated HLA–A2/peptide pentamer was purchased from ProImmune (Oxford, UK), multimerized in-house using SA-PE or SA-APC (Life Technologies), and utilized to stain antigen-specific T cells as described previously^25^,^26^,^27^,^59^. TCR-transduced Jurkat 76 or Jurkat 76/CD8 cells (1 × 10^5^) were incubated with 2 μg/ml A2/MART1 or A2/HIV multimer for 30 minutes at room temperature and costained with anti-CD3 or anti-CD8 mAb for 15 minutes at 4 °C. A2/HIV multimer was always used as a control. Structural avidity was determined by staining with graded concentrations of A2/MART1 multimer as reported previously^18^. The EC_50_ was defined as the concentration of multimer required to achieve 50% of the maximal multimer staining.

### HLA/peptide monomer staining

TCR-transduced Jurkat 76 or Jurkat 76/CD8 cells (1 × 10^5^) were incubated with 50 μg/ml biotinylated, non-multimerized A2/MART1 monomer or A2/CYP1B1 monomer, which was generated in-house, for 1 hour at room temperature^23^,^24^,^27^,^58^,^60^. After 3 washes for the removal of unbound monomeric pMHC complexes, the T cells were costained with anti-biotin and anti-CD3 or anti-CD8 mAb for 15 minutes at 4 °C. A2/CYP1B1 monomer was always used as a control.

### Cytokine ELISPOT analysis

IL-2 and IFN-γ ELISPOT assays were conducted as described elsewhere^25^,^27^. For the IL-2 ELISPOT assay, PVDF plates (Millipore, Bedford, MA) were coated with capture mAb (SEL002; R&D Systems, Minneapolis, MN). T cells were incubated with 2 × 10^4^ stimulator cells for 20–24 hours at 37 °C. The plates were washed and incubated with biotin-conjugated detection mAb (SEL002; R&D Systems). After washing, alkaline phosphatase-conjugated streptavidin (Jackson ImmunoResearch) was added. The plates were washed and incubated with NBT/BCIP (nitroblue tetrazolium/5-bromo-4-chloro-3-indolyl phosphate; Promega), and IL-2 spots were developed. For the IFN-γ ELISPOT assay, PVDF plates (Millipore, Bedford, MA) were coated with capture mAb (1D1K; MABTECH, Mariemont, OH). T cells were incubated with 2 × 10^4^ stimulator cells for 20–24 hours at 37 °C. The plates were washed and incubated with biotin-conjugated detection mAb (7-B6-1; MABTECH). HRP-conjugated SA (Jackson ImmunoResearch) was then added, and IFN-γ spots were developed. The reaction was stopped by rinsing thoroughly with cold tap water. ELISPOT plates were scanned and counted using an ImmunoSpot plate reader and ImmunoSpot version 5.0 software (Cellular Technology Limited, Shaker Heights, OH). Functional avidity was tested using T2 cells pulsed with graded concentrations of wild-type A2/MART1_27–35_ peptide as stimulators in ELISPOT assays as reported previously^18^,^26^. The EC_50_ was defined as the concentration of peptide required to achieve 50% of the maximal response.

### Statistical analysis

Statistical analysis was performed using GraphPad Prism 6.0 software. Unpaired two-tailed Welch’s t tests were used for two-sample comparisons (Figs 4b and 5b,d, Supplementary Fig. 3c, and Supplementary Fig. 6). Two-tailed paired t tests were used for two-sample comparisons (Fig. 4c). P values of <0.05 were considered significant. No statistical method was used to predetermine sample size. The investigators were not blinded to allocation during the experiments or outcome assessment. The experiments were not randomized.

## Additional Information

**How to cite this article**: Nakatsugawa, M. *et al.* CD4^+^ and CD8^+^ TCRβ repertoires possess different potentials to generate extraordinarily high-avidity T cells. *Sci. Rep.* **6**, 23821; doi: 10.1038/srep23821 (2016).

## Supplementary Material

Supplementary Information

## Figures and Tables

**Table 1:** Functional and structural avidities of the A2/MART1 TCRs.

Clone	T cells derived from	TRBV	CDR3β	TRBJ	Structural avidity* without CD8	Structural avidity with CD8	EC_50_(μg/ml)	EC_50_(μg/ml)
							Functional avidity† without CD8	Functional avidity with CD8
Cl.7E	CD4	27	CASSRDFGNTIYF	1–3	0.010	0.006	0.102	0.071
Cl.3P	CD4	5–1	CASSLTGGYGYTF	1–2	0.013	0.012	0.116	0.058
Cl.830	CD8	27	CASSLGGAYEQYF	2–7	0.013	0.008	0.056	0.036
Cl.8H	CD4	27	CASSPLGAMEQYF	2–7	0.014	0.008	0.063	0.040
Cl.1606	CD8	27	CASSLLGSYEQYF	2–7	0.015	0.008	0.031	0.031
Cl.7Q	CD4	27	CASSPYMMNTEAFF	1–1	0.016	0.009	0.065	0.043
Cl.1593	CD8	27	CASGNNQPQHF	1–5	0.016	0.008	0.104	0.047
Cl.9J	CD4	5–1	CASSWTGDGYTF	1–2	0.018	0.010	0.075	0.071
Cl.2J	CD4	27	CAASMGQGFGEQFF	2–1	0.018	0.008	0.100	0.072
Cl.2E	CD4	27	CASSWDWGNIQYF	2–4	0.019	0.014	0.070	0.056
Cl.11E	CD4	2	CASSVMAPLHF	1–6	0.022	0.011	0.121	0.074
DMF5‡	CD8	6–4	CASSLSFGTEAFF	1–1	0.022	0.013	0.363	0.102
Cl.4D	CD4	5–1	CASSWAGTGSEQYF	2–7	0.030	0.022	0.179	0.073
Cl.6X	CD4	2	CATGVTDTQYF	2–3	0.039	0.018	0.150	0.083
Cl.10V	CD4	5–1	CASSLQGANGELFF	2–2	0.043	0.033	0.405	0.140
Cl.6B	CD4	2	CASSEVAWQFF	2–1	0.044	0.013	0.600	0.120
Cl.1086	CD8	27	CASSLHGPGGYTF	1–2	0.048	0.013	0.554	0.076
Cl.3J	CD4	5–1	CASSLMGTEAFF	1–1	0.050	0.025	1.451	0.263
Cl.1599	CD8	27	CASSFLGAMAEAFF	1–1	0.069	0.018	0.053	0.032
Cl.6I	CD4	2	CATGRGATQYF	2–3	0.070	0.020	0.665	0.125
Cl.788	CD8	27	CASGPSYEQYF	2–7	0.071	0.013	0.880	0.121
Cl.794	CD8	27	CASSLLGDYGYTF	1–2	0.082	0.018	0.067	0.043
Cl.11C	CD4	2	CASDEGFGYTF	1–2	0.108	0.018	2.460	0.292
Cl.1574	CD8	27	CASSPWERINTEAFF	1–1	0.110	0.021	0.076	0.052
Cl.758	CD8	27	CASSPRLAGDGELFF	2–2	1.610	0.041	0.342	0.096
Cl.523	CD8	27	CASGSYEQYF	2–7	n.m	0.064	n.m	0.748
Cl.413	CD8	27	CASSVFGGDMGEKLFF	1–4	n.m	0.303	n.m	2.337

^*^Structural avidity, expressed as EC_50_ in μg/ml, was defined as the concentration of A2/MART1 multimer required to achieve 50% of maximal multimer staining.

^†^Functional avidity, expressed as EC_50_ in μg/ml, was defined as the concentration of peptide required to achieve 50% of maximal response.

^‡^DMF5 is a high affinity A2/MART1 TCR reported by Johnson *et al.*^28^ n.m, not measurable.
