# Supplementary material for: CD4+ and CD8+ TCRβ repertoires possess different potentials to generate extraordinarily high-avidity T cells
Source: Sci Rep. 2016 Mar 31;6:23821. doi: 10.1038/srep23821 (PMC4814874; doi:10.1038/srep23821)
Supplement: Supplementary Information [file srep23821-s1.pdf]

Supplemental information for:

CD4$^+$ and CD8$^+$ TCRβ repertoires possess different potentials to generate extraordinarily high-avidity T cells

Munehide Nakatsugawa[1], Muhammed A. Rahman[1], Yuki Yamashita[1], Toshiki Ochi[1], Piotr Wnuk[1, 2], Shinya Tanaka[1, 3], Kenji Chamoto[1], Yuki Kagoya[1], Kayoko Saso[1], Tingxi Guo[1, 2], Mark Anczurowski[1, 2], Marcus O. Butler[1, 4], and Naoto Hirano[1, 2]

[1]Tumor Immunotherapy Program, Campbell Family Institute for Breast Cancer Research, Campbell Family Cancer Research Institute, Princess Margaret Cancer Centre, University Health Network, Toronto, ON M5G 2M9, Canada; [2]Department of Immunology, University of Toronto, Toronto, ON M5S 1A8, Canada; [3]Takara Bio, Inc., Kusatsu, Shiga 525-0058, Japan; [4]Department of Medicine, University of Toronto, Toronto, ON M5S 1A8, Canada.

Author information

Correspondence and requests for materials should be addressed to N.H. (naoto.hirano@utoronto.ca).

Supplementary Figures 1-6

Supplementary Tables 1-4

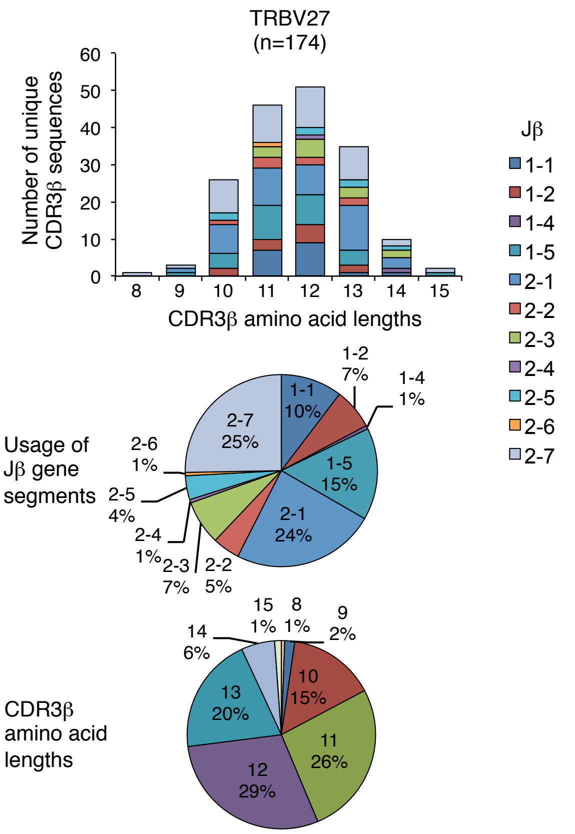

Supplementary Figure 1 | Highly heterogeneous TRBV27 TCRβ chains expressed in both HLA-A2^{+} and A2^{-} CD8^{+} T cells recognize A2/MART1 when paired with SIG35α. SIG35α/ΔNGFR-transduced CD8^{+} T cells were stimulated with aAPC pulsed with A2/MART1 peptide. ΔNGFR^{+} A2/MART1 multimer^{+} CD8^{+} T cells were collected by fluorescence activated cell sorting (>99% purity), and their TRBV27 CDR3β regions were amplified by PCR and sequenced after cloning. The number of unique CDR3β sequences (top), the relative usage of Jβ gene segments (middle), and the CDR3β amino acid lengths (bottom) are depicted.

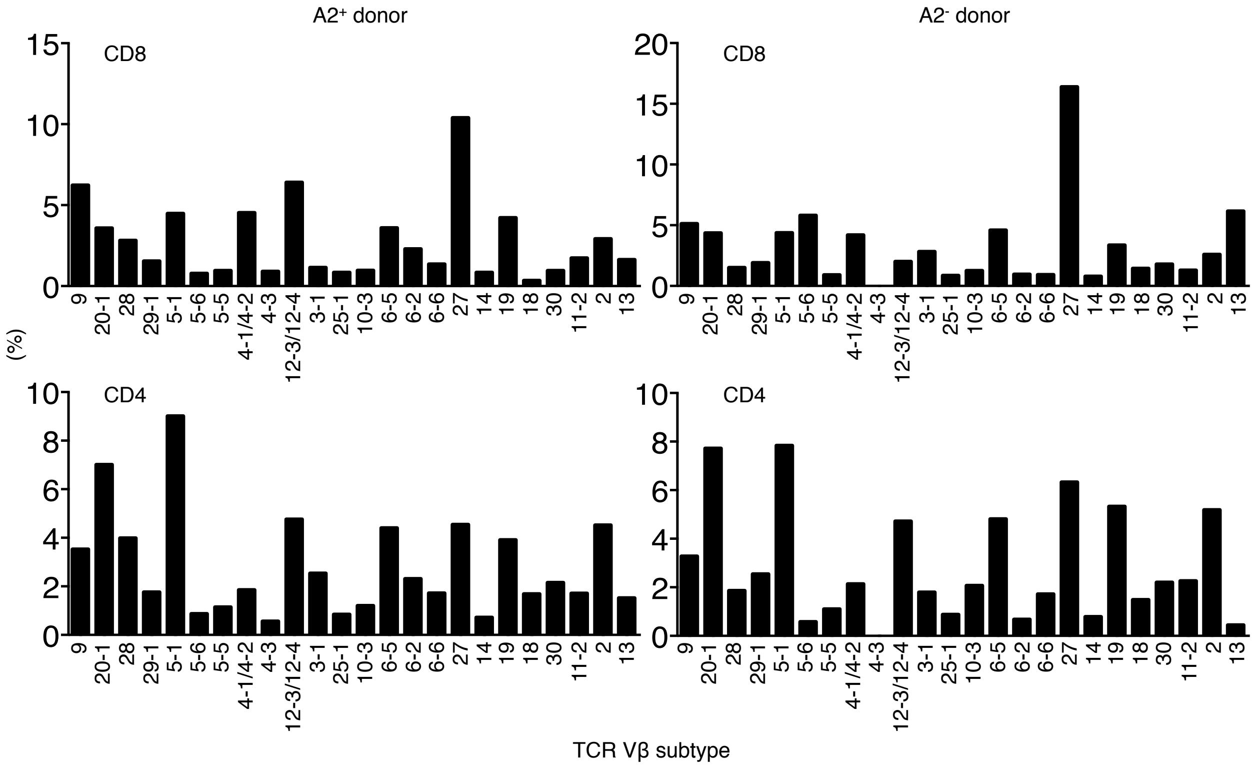

Supplementary Figure 2 | TCR Vβ subtype analysis of overall SIG35α-transduced $CD4^+$ and $CD8^+$ T cells in the HLA-$A2^+$ and $A2^-$ donors. SIG35α/ΔNGFR-transduced peripheral $CD8^+$ (top) and $CD4^+$ (bottom) T cells from the HLA-$A2^+$ and $A2^-$ donors were stimulated with aAPC pulsed with A2/MART1 peptide twice and stained with A2/MART1 multimer, mAbs for TCR Vβ subtypes, anti-CD4 or CD8 mAb and anti-NGFR mAb. The percentage of overall SIG35α-transduced $CD8^+$ (top) and $CD4^+$ (bottom) T cells expressing each subtype is shown. The data shown are gated on $ΔNGFR^+$ cells.

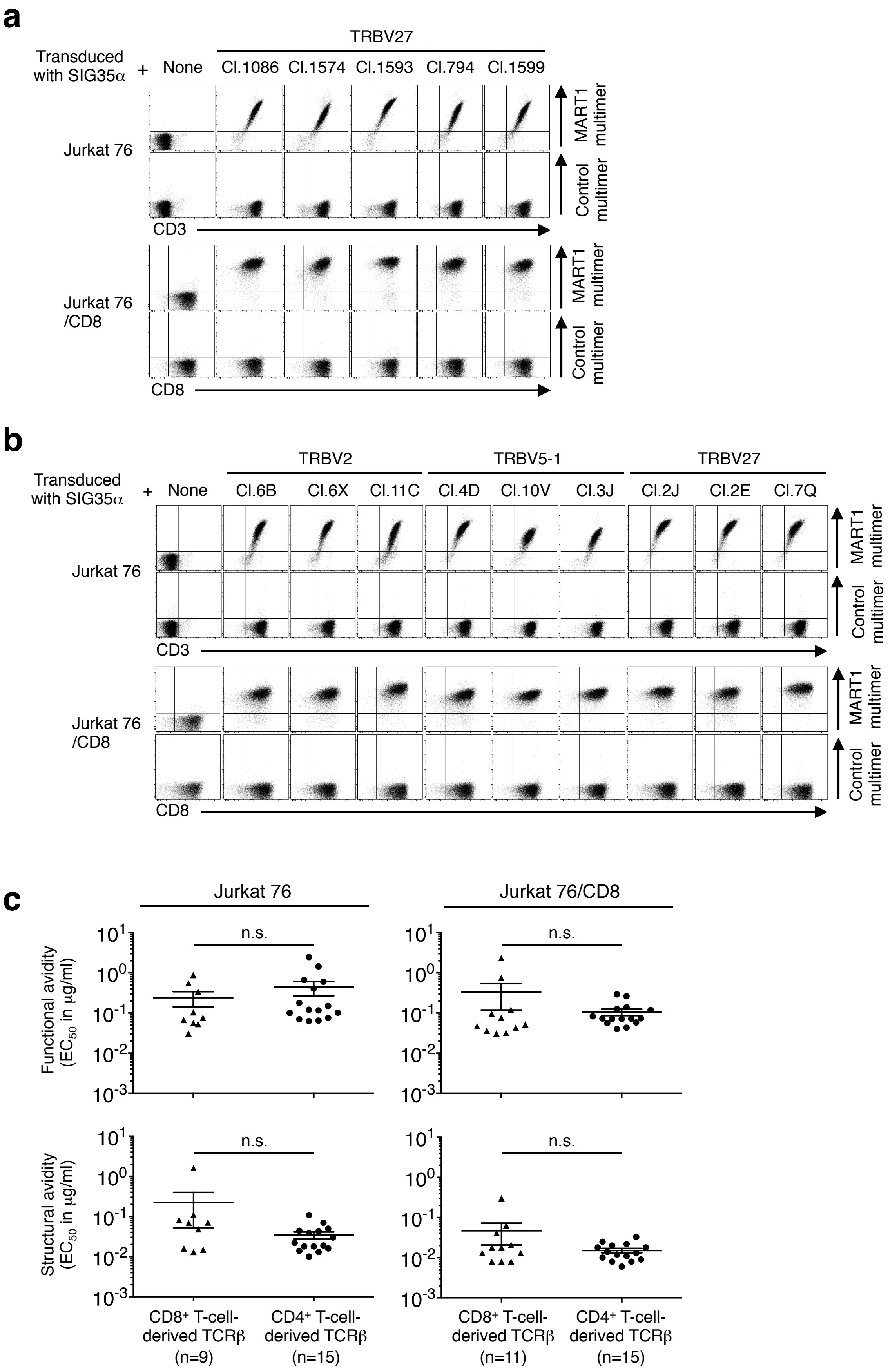
a
TRBV27
Transduced with SIG35α
+
None
Cl.1086
Cl.1574
Cl.1593
Cl.794
Cl.1599
Jurkat 76
MART1 multimer
Control multimer
CD3
Jurkat 76 /CD8
MART1 multimer
Control multimer
CD8
b
TRBV2
TRBV5-1
TRBV27
Transduced with SIG35α
+
None
Cl.6B
Cl.6X
Cl.11C
Cl.4D
Cl.10V
Cl.3J
Cl.2J
Cl.2E
Cl.7Q
Jurkat 76
MART1 multimer
Control multimer
CD3
Jurkat 76 /CD8
MART1 multimer
Control multimer
CD8
c
Jurkat 76
Jurkat 76/CD8
n.s.
n.s.
n.s.
n.s.
Functional avidity (EC50 in μg/ml)
Structural avidity (EC50 in μg/ml)
10^1
10^0
10^-1
10^-2
10^-3
CD8+ T-cell-derived TCRβ (n=9)
CD4+ T-cell-derived TCRβ (n=15)
CD8+ T-cell-derived TCRβ (n=11)
CD4+ T-cell-derived TCRβ (n=15)

Supplementary Figure 3 | Structural avidity measured using a multimeric complex as well as functional avidity are comparable between transfectants expressing A2/MART1 TCRβ chains derived from $CD4^+$ and $CD8^+$ T cells. Jurkat 76 and Jurkat 76/CD8 cells were transduced with one of eleven clonotypic TRBV27 TCRβ chains cloned from SIG35α^+ A2/MART1 $CD8^+$ T cells or five clonotypic TCRβ chains each for TRBV2, TRBV5-1 and TRBV27 cloned from SIG35α^+ A2/MART1 $CD4^+$ T cells along with SIG35α or DMF5αβ chains. (**a, b**) All Jurkat 76 and Jurkat 76/CD8 transfectants were stained with 2 μg/ml A2/MART1 or control multimer along with anti-CD3 mAb or anti-CD8 mAb. Data for multimer staining of 6 representative $CD8^+$ T-cell-derived TCRβ transfectants, 6 representative $CD4^+$ T-cell-derived TCRβ transfectants, and DMF5 TCR transfectants are shown in Fig. 3a. Data for multimer staining of the remaining 5 $CD8^+$ T-cell-derived TCRβ transfectants (**a**) and 9 $CD4^+$ T cell-derived TCRβ transfectants (**b**) in the presence or absence of CD8 coreceptor expression are shown. (**c**) Functional and structural avidity were compared in Jurkat 76 transfectants expressing $CD8^+$ or $CD4^+$ T-cell-derived TCRβ chains in the absence (left) or presence (right) of CD8 coexpression. The data represent the means ± SD in each group. Note that functional and structural avidity could not be calculated for Jurkat 76-expressing cl. 413 and 523 with low reactivity. The structural and functional avidity data for all transfectants are summarized in Table 1. n.s., not significant, two-tailed Welch's t test. All data shown are representative of two independent experiments.

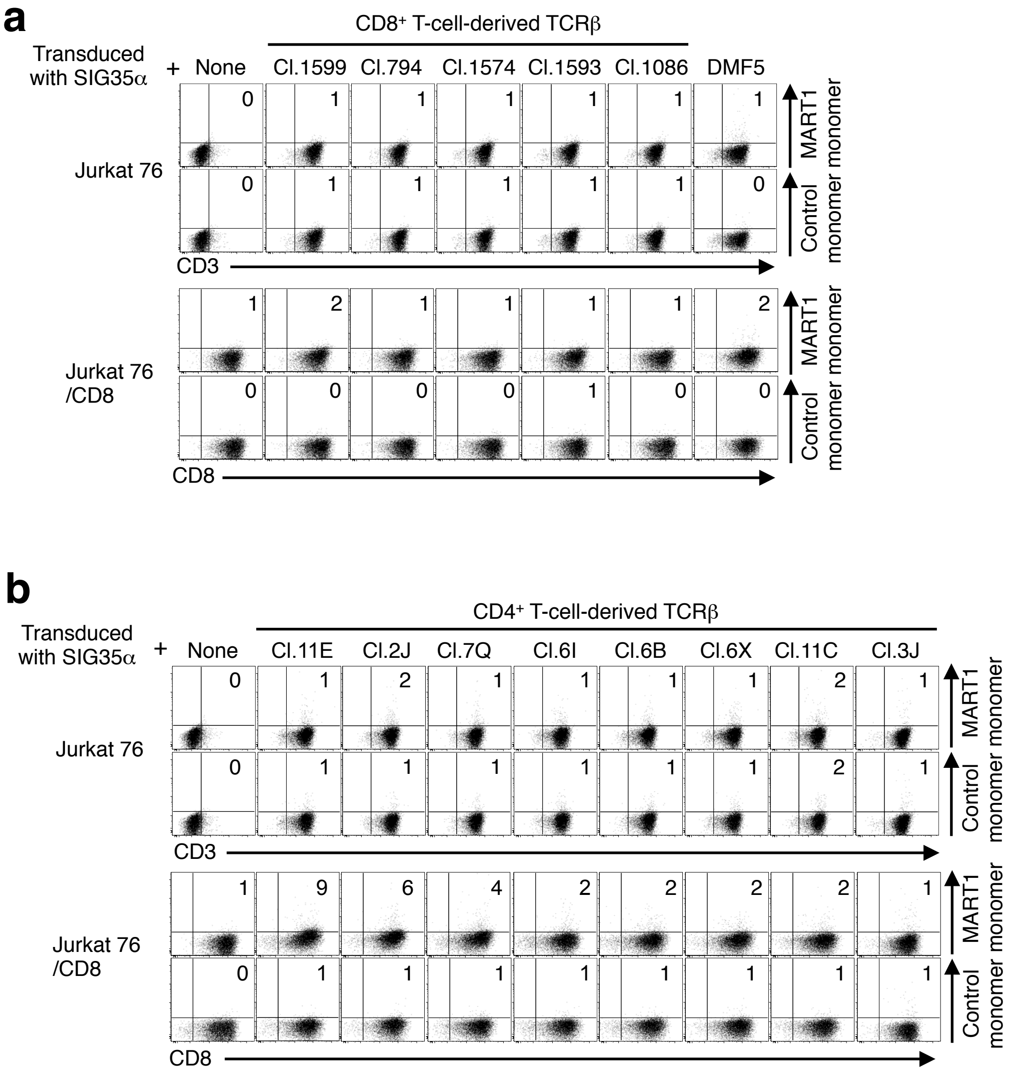

Supplementary Figure 4 | Monomer staining of Jurkat 76 transfectants expressing A2/MART1 TCRβ chains derived from CD8⁺ and CD4⁺ T cells in the presence or absence of CD8 coreceptor expression. (**a, b**) Jurkat 76 and Jurkat 76/CD8 transfectants were stained with 50 μg/ml non-multimerized A2/MART1 or control monomer along with anti-CD3 mAb or anti-CD8 mAb. Data for monomer staining of 6 representative CD8⁺ T-cell-derived TCRβ transfectants and 7 representative CD4⁺ T-cell-derived TCRβ transfectants are shown in Fig. 5a. Data for monomer staining of the remaining 5 CD8⁺ T-cell-derived TCRβ transfectants and DMF5 TCR transfectants (**a**) and 8 CD4⁺ T-cell-derived TCRβ transfectants (**b**) in the presence or absence of CD8 coexpression are shown. The data shown are representative of three independent experiments.

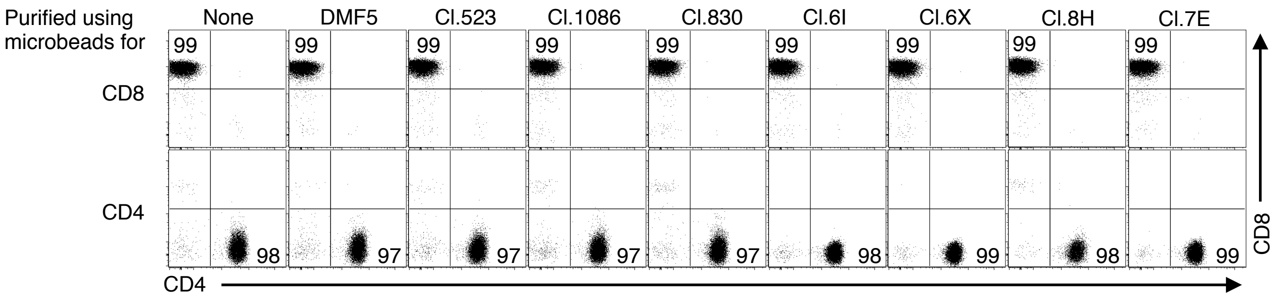

Supplementary Figure 5 | Purity of CD8$^+$ and CD4$^+$ T cells after purification using microbeads. Peripheral T cells were retrovirally transduced with TCRβ (cl. 523, 1086, 830, 6I, 6X, 8H, or 7E) genes along with SIG35α or DMF5 TCR. CD8$^+$ and CD4$^+$ T cells were purified using microbeads and stained with anti-CD8 mAb and anti-CD4 mAb.

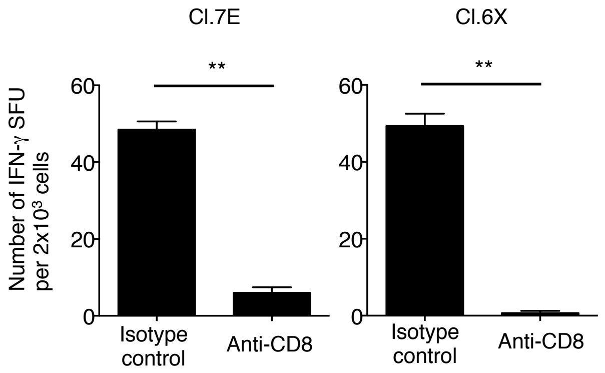

Supplementary Figure 6 | Anti-CD8 mAb blocks self-reactivity in CD8$^+$ T cells expressing cl. 7E and 6X TCRβ chains. Peripheral CD8$^+$ T cells were retrovirally transduced with cl. 7E and 6X TCRβ along with SIG35α. IFN-γ ELISPOT assay was performed using the CD8$^+$ T cells expressing cl. 7E and 6X as responder cells in the presence of 10 μg/ml isotype control or anti-CD8 mAb (clone DK-25) [2]. A2$^+$ MART1$^-$ A375 cells were used as stimulator cells. The data represent the means ± SD in each group. **$p<0.01$, two-tailed Welch's t test.

Supplementary Table 1

TCRβ chains isolated from SIG35α+ A2/MART1 CD8+ T cells

Clone	TRBV	CDR3β	TRBJ	Clone	TRBV	CDR3β	TRBJ	Clone	TRBV	CDR3β	TRBJ
031	27	CASSLDSVNTEAFF	1-1	007	27	CASSLSGQGNEQFF	2-1	750	27	CASSFLLPTDTQYF	2-3
376	27	CASSLFVNTEAFF	1-1	013	27	CASSPLAGVNEQFF	2-1	780	27	CASSLTSGFTDTQYF	2-3
378	27	CASSLGPNTEAFF	1-1	047	27	CASLTVYNEQFF	2-1	782	27	CASSLAGGLTDTQYF	2-3
380	27	CASSLSGGQGEAFF	1-1	092	27	CASSLIGYEQFF	2-1	787	27	CASSIWTGTDTQYF	2-3
434	27	CASGWQGVTEAFF	1-1	098	27	CASSPQGAGVGEQFF	2-1	1609	27	CASSFLGYTDTQYF	2-3
435	27	CASSFGANTEAFF	1-1	108	27	CASSFTGNEQFF	2-1	666	27	CASSSGQGQNIQYF	2-4
457	27	CASSFLGDNTEAFF	1-1	156	27	CASSSGATAGNEQFF	2-1	050	27	CASSLYVGRQETQYF	2-5
522	27	CASSLAHTYTEAFF	1-1	321	27	CASSLSVGTINEQFF	2-1	136	27	CASSLLGAEETQYF	2-5
558	27	CASSPSGTVGAFF	1-1	366	27	CASSPLAGQNHEQFF	2-1	377	27	CASSQGLETQYF	2-5
571	27	CASSPQTGGTEAFF	1-1	369	27	CATGLAAYNEQFF	2-1	393	27	CASSRTTGQQETQYF	2-5
641	27	CASSLSPVGEAFF	1-1	389	27	CASSGDYNEQFF	2-1	513	27	CASSLYGGGSGETQYF	2-5
642	27	CASSFIGSTEAFF	1-1	509	27	CASSLSSGDFYNEQFF	2-1	781	27	CASSPTGGDETQYF	2-5
753	27	CASSLNGQAGEAFF	1-1	514	27	CASSPPPGGGADEQFF	2-1	1614	27	CASSPLGGTQYF	2-5
796	27	CASSPTGFTSNTEAFF	1-1	518	27	CASSLVGNEQFF	2-1	747	27	CASSLSGANVLTF	2-6
990	27	CASSPMWGPTEAFF	1-1	524	27	CASSLSGYNEQFF	2-1	005	27	CASSSTGYEQYF	2-7
1574*	27	CASSPWERINTEAFF	1-1	527	27	CASSFISGGNEQFF	2-1	006	27	CASSLTGTGGEQYF	2-7
1592	27	CASSLLGMIHGAFF	1-1	610	27	CASSLNPYNEQFF	2-1	017	27	CASSPHPPEQYF	2-7
1599*	27	CASSFLGAMAEAFF	1-1	624	27	CASSPGTEQFF	2-1	076	27	CASSPSGYEQYF	2-7
044	27	CASSLGGMGYTF	1-2	634	27	CASSLWTSGSNEQFF	2-1	094	27	CASSFGDRTYEQYF	2-7
418	27	CASSVSGQLFYGYTF	1-2	637	27	CASSYDSPHEQFF	2-1	103	27	CASSTGVNEQYF	2-7
419	27	CASSLTVTHYGYTF	1-2	691	27	CASSNTGYNEQFF	2-1	112	27	CASSFGGTYEQYF	2-7
431	27	CASSLSGPNYGYTF	1-2	749	27	CASSLYGAPDEQFF	2-1	120	27	CASGWGPYEQYF	2-7
433	27	CASSLSPGDFGYTF	1-2	755	27	CASSSQYNEQFF	2-1	130	27	CASSLSPASYEQYF	2-7
507	27	CASSLYPGSYGYTF	1-2	763	27	CASSLGLAGTNEQFF	2-1	165	27	CASSFGGLEQYF	2-7
566	27	CASSLSFNPDYGYTF	1-2	766	27	CASSLVLSSYNEQFF	2-1	320	27	CASSPSGGSSYEQYF	2-7
578	27	CASSWSGGNYGYTF	1-2	773	27	CASSPGFHEQFF	2-1	322	27	CASSLFAGQVYEQYF	2-7
599	27	CASSLTGYGYTF	1-2	777	27	CASSRTVTGEQFF	2-1	337	27	CASSLSPGQVGEQYF	2-7
628	27	CASSLTLFGGYTF	1-2	783	27	CASSLGPVYEQFF	2-1	385	27	CASSDTGVREQYF	2-7
794*	27	CASSLLGDYGYTF	1-2	784	27	CASSLIFGGLDEQFF	2-1	394	27	CASSWTSGNPEQYF	2-7
1086*	27	CASSLHGPGGYTF	1-2	791	27	CASSLLGSHEQFF	2-1	399	27	CASSPSAGLSYEQYF	2-7
413*	27	CASSVFGGDMGEKLFF	1-4	792	27	CASSLSALVEQFF	2-1	407	27	CASSLAGGSYEQYF	2-7
073	27	CASSTTLNQPQHF	1-5	793	27	CASSFAGNEQFF	2-1	512	27	CASSWAGSYEQYF	2-7
373	27	CASSLWGAGNQPQHF	1-5	823	27	CASSLNIGGAGEQFF	2-1	520	27	CASSAGGPGYEQYF	2-7
381	27	CASSGGNQPQHF	1-5	825	27	CASSPISRLDEQFF	2-1	523*	27	CASGSYEQYF	2-7
411	27	CASSDRGYQPQHF	1-5	828	27	CASSLSLSYNEQFF	2-1	608	27	CASSSTGVGQYF	2-7
436	27	CASSLSGVQPQHF	1-5	836	27	CASSLLLVGDEQFF	2-1	632	27	CASSPGQGTYEQYF	2-7
442	27	CASSDLTGPNQPQHF	1-5	1014	27	CASSFLAGGLNEQFF	2-1	635	27	CASSPYSGSPSLEQYF	2-7
444	27	CASSFTGGQPQHF	1-5	1029	27	CASRPTSGRLDEQFF	2-1	644	27	CASSPLGGGYEQYF	2-7
445	27	CASSLSSINQPQHF	1-5	1031	27	CASSPSVGLAVNEQFF	2-1	685	27	CASSQGGGYEQYF	2-7
446	27	CASSLGGARPQHF	1-5	1603	27	CASSFIGKYNEQFF	2-1	693	27	CASSPASGAVYEQYF	2-7
448	27	CASSLSVTFGQPQHF	1-5	1604	27	CASSLTSWAGNEQFF	2-1	746	27	CASSFGTGVEQYF	2-7
451	27	CASSLVGQPQHF	1-5	1608	27	CASSFLGADEQFF	2-1	748	27	CASSLFSGNSYEQYF	2-7
452	27	CASSFPNQPQHF	1-5	095	27	CASSLMSVAGELFF	2-2	752	27	CASSPSTYRGYEQYF	2-7
453	27	CASSLGQVNQPQHF	1-5	107	27	CASSFGTGELFF	2-2	754	27	CASSYRLGFYEQYF	2-7
454	27	CASSLSSNQPQHF	1-5	374	27	CASSWTGTGELFF	2-2	767	27	CASSFGGGGTGLYEQYF	2-7
508	27	CASSLGQGGQPQHF	1-5	388	27	CASSLGATGELFF	2-2	768	27	CASSLHSGSSYEQYF	2-7
515	27	CASSLYSGNYQPQHF	1-5	398	27	CASSLSITGELFF	2-2	770	27	CASSLGGFYEQYF	2-7
519	27	CASSSSNQPQHF	1-5	667	27	CASSGLANAGELFF	2-2	775	27	CASGLPVGEQYF	2-7
573	27	CASSIEGAGQPQHF	1-5	758*	27	CASSPRLAGDGELFF	2-2	788*	27	CASGPSYEQYF	2-7
575	27	CASSPGAGGPQHF	1-5	765	27	CASSLGPPGAGELFF	2-2	819	27	CASSSLGSYEQYF	2-7
579	27	CASSLNSGGLSNQPQHF	1-5	102	27	CASSPTSGGGGTQYF	2-3	830*	27	CASSLGGAYEQYF	2-7
592	27	CASSLSPTDSLQHF	1-5	124	27	CASSYGGSDTQYF	2-3	833	27	CASSPTSGYYEQYF	2-7
598	27	CASSIGTGQQPQHF	1-5	152	27	CASSLSPSGGTDTQYF	2-3	896	27	CASSPSVGTEAYEQYF	2-7
790	27	CASSFGQGNQPQHF	1-5	331	27	CASGTWVLDTQYF	2-3	898	27	CASSALPPEQYF	2-7
801	27	CASSLGGAQPQHF	1-5	406	27	CASSFLGQGDTDTQYF	2-3	912	27	CASSFGTAYEQYF	2-7
993	27	CASSFLGAGQPQHF	1-5	517	27	CASSLVGGADTQYF	2-3	1022	27	CASSLYPSGVYEQYF	2-7
1593*	27	CASGNNQPQHF	1-5	609	27	CASSSGASTDTQYF	2-3	1606*	27	CASSLLGSYEQYF	2-7
1597	27	CASSLFNSQPQHF	1-5	687	27	CASSPLASDTQYF	2-3	1612	27	CASSLYSNPGEQYF	2-7

174 unique TRBV27 TCRβ chains were isolated from SIG35α+ A2/MART1 CD8+ T cells. CDR3β sequences are shown.
*Randomly selected 11 clones which were reconstituted on Jurkat 76 or Jurkat 76/CD8.

Supplementary Table 2

TCRβ chains isolated from SIG35α+ A2/MART1 CD4+ T cells

Clone	TRBV	CDR3β	TRBJ
TRBV2			
11M	2	CAGAGGPEAFF	1-1
11F	2	CASTLGPEAFF	1-1
11C*	2	CASDEGFGYTF	1-2
5Q	2	CASSDVGSVNYGYTF	1-2
5A	2	CASSEAATIYF	1-3
11V	2	CASSEAAAQHF	1-5
11E*	2	CASSVMAPLHF	1-6
6O	2	CASSVAVEQFF	2-1
12C	2	CASSEAVTKFF	2-1
6B*	2	CASSEVAWQFF	2-1
12G	2	CASSYSGVEQFF	2-1
6J	2	CASSESDTQYF	2-3
6X*	2	CATGVTDTQYF	2-3
6I*	2	CATGRGATQYF	2-3
TRBV5-1			
3J*	5-1	CASSLMGTEAFF	1-1
3Q	5-1	CASSLVGGEAFF	1-1
3H	5-1	CASSLTGGTEAFF	1-1
9Q	5-1	CASSFGAGGEAFF	1-1
9I	5-1	CASSLAMGATEAFF	1-1
3G	5-1	CASSLVGDGYTF	1-2
9J*	5-1	CASSWTGDGYTF	1-2
9B	5-1	CASSLVGEGYTF	1-2
3P*	5-1	CASSLTGGYGYTF	1-2
9G	5-1	CASSLGAGGGYTF	1-2
3B	5-1	CASSLAGGSGYTF	1-2
10V*	5-1	CASSLQGANGELFF	2-2
10F	5-1	CASSLQGVAQYF	2-3
4X	5-1	CASSFDGAKNIQYF	2-4
4E	5-1	CASSQEAGAQETQYF	2-5
10P	5-1	CASSLVGYEQYF	2-7
10W	5-1	CASSLGGVEQYF	2-7
10X	5-1	CASSLSGNEQYF	2-7
4K	5-1	CASSHGGNEQYF	2-7
10C	5-1	CASSLSSAQQYF	2-7
4B	5-1	CASSFAGGYEQYF	2-7
4D*	5-1	CASSWAGTGSEQYF	2-7
TRBV27			
1G	27	CASSSPRAEAFF	1-1
7Q*	27	CASSPYMMNTEAFF	1-1
1S	27	CASSLTGHGYTF	1-2
7C	27	CASSLSQTGGPATF	1-2
7E*	27	CASSRDFGNTIYF	1-3
1M	27	CASSFLGQPQHF	1-5
7A	27	CASSFGGGQPQHF	1-5
1O	27	CASSLWATGQPQHF	1-5
1W	27	CASSLSGGAGPQHF	1-5
1B	27	CASSLGSGANQPQHF	1-5
7W	27	CASSLWFGSNQPQHF	1-5
8E	27	CASSWGASGGQFF	2-1
8G	27	CASSLSPTVEQFF	2-1
2J*	27	CAASMGQGFGEQFF	2-1
2L	27	CASSLGTGGGEQFF	2-1
8C	27	CASSFGLAGGSEFF	2-1
8U	27	CASSLLGRDNEQFF	2-1
2O	27	CASSPGLAAGHTQYF	2-3
2E*	27	CASSWDWGNIQYF	2-4
8M	27	CASSFLGGPLQYF	2-4
2A	27	CASSPAAIMQTQYF	2-5
2I	27	CASSPLGGVETQYF	2-5
8H*	27	CASSPLGAMEQYF	2-7
2F	27	CASSLGGGGYEQYF	2-7
2H	27	CASSLSLVGWEQYF	2-7
8A	27	CASSLSPAGGEQYF	2-7

14 TRBV2, 22 TRBV5-1 and 26 TRBV27 independent clonotypic TCRβ chains were isolated from SIG35α+ A2/MART1 CD4+ T cells. CDR3β sequences are shown.
*Randomly selected 5 each TRBV clones which were reconstituted on Jurkat 76 or Jurkat 76/CD8.

Supplementary Table 3

Sequencing results of TCRβ chains isolated from SIG35α^+ A2/MART1 CD4$^+$ T cells

Vβ subtypes	$A2^+$ donor		$A2^-$ donor		Number of shared clonotypes
	Number of unique clonotypes	Number of isolates sequenced	Number of unique clonotypes	Number of isolates sequenced	
TRBV2	7	42	7	36	0
TRBV5-1	11	19	11	31	0
TRBV27	13	29	13	30	0

Supplementary Table 4

Sequences of MART1-related peptides*

Protein	Peptide sequence
KIAA0935	RVTDEAGHPV
cMOAT2	NVADIGLHDV
SLC1A1	VLTGLAIHSI
P47	RISDIRLFIV
Prostaglandin transporter (PGT)	LLAGIGTVPI
MOAT-C	RISDIGLADL
KIAA0735	LISGIGIGGA
Hypothetical 20 kD protein (HP)	RISAIILHPN
Endothelin-1 receptor (ETR)	RVQGIGIPLV
G-protein coupled receptor (RE2)	RITDLGLSPH
IGHG1	RLSELAIFGV
Monocarboxylate transporter 8 (MT8)	AVAFIGLHTS
MRP3	NVADIGFHDV

*MART1-related peptides reported by Dutoit et al. [1]

Supplementary References

1. Dutoit V*, et al.* Degeneracy of antigen recognition as the molecular basis for the high frequency of naive A2/ Melan-a peptide multimer(+) CD8(+) T cells in humans. *J Exp Med* **196**, 207-216 (2002).

2. Wooldridge L, et al. Anti-CD8 antibodies can inhibit or enhance peptide-MHC class I (pMHCI) multimer binding: this is paralleled by their effects on CTL activation and occurs in the absence of an interaction between pMHCI and CD8 on the cell surface. *J Immunol* **171**, 6650-6660 (2003).
